# Reading into neuronal oscillations in the visual system: implications for developmental dyslexia

**DOI:** 10.3389/fnhum.2013.00811

**Published:** 2013-11-27

**Authors:** Trichur R. Vidyasagar

**Affiliations:** ^1^Visual and Cognitive Neuroscience Laboratory, Department of Optometry and Vision Sciences, University of MelbourneParkville, VIC, Australia; ^2^Melbourne Neuroscience Institute, University of MelbourneParkville, VIC, Australia

**Keywords:** developmental dyslexia, neuronal oscillations, posterior parietal cortex, primary visual cortex, top-down attention, reading

## Abstract

While phonological impairments are common in developmental dyslexia, there has recently been much debate as to whether there is a causal link between the phonological difficulties and the reading problem. An alternative suggestion has been gaining ground that the core deficit in dyslexia is in visual attentional mechanisms. If so, the visual aetiology may be at any of a number of sites along the afferent magnocellular pathway or in the dorsal cortical stream that are all essential for a visuo-spatial attentional feedback to the primary visual cortex. It has been suggested that the same circuits and pathways of top-down attention used for serial visual search are used for reading. Top-down signals from the dorsal parietal areas to primary visual cortex serially highlight cortical locations representing successive letters in a text before they can be recognized and concatenated into a word. We had shown in non-human primates that the mechanism of such a top-down feedback in a visual attention task uses synchronized neuronal oscillations at the lower end of the gamma frequency range. It is no coincidence that reading graphemes in a text also happens at the low gamma frequencies. The basic proposal here is that each cycle of gamma oscillation focuses an attentional spotlight on the primary visual cortical representation of just one or two letters before sequential recognition of letters and their concatenation into word strings. The timing, period, envelope, amplitude, and phase of the synchronized oscillations modulating the incoming signals in the striate cortex would have a profound influence on the accuracy and speed of reading. Thus, the general temporal sampling difficulties in dyslexic subjects may impact reading not necessarily by causing phonological deficits, but by affecting the spatio-temporal parsing of the visual input within the visual system before these signals are used for letter and word recognition.

## Introduction

For nearly three decades, the dominant model to explain the reading difficulties experienced by those with developmental dyslexia (DD) was one that was phonologically based (Bradley and Bryant, 1983; Goswami and Bryant, 1990; Stanovich, 1998; Shaywitz and Shaywitz, 2005; Ziegler and Goswami, 2005; Goswami, 2011). This was supported not only by the profound deficits in phonological skills and in phonemic awareness commonly found to be associated with dyslexia, but also by the findings of temporal processing difficulties in the auditory system that provided a possible neuronal basis for the phonological theory (Tallal, 1980; Ahissar et al., 2000; Temple et al., 2000; Breier et al., 2001). Recently, these findings were integrated with a host of studies on neuronal oscillatory mechanisms that are relevant for temporal sampling of speech and were applied to DD in a model termed the “temporal sampling framework” (TSF) by Goswami (2011). This was a fresh new approach in the field and brought a sound neurophysiological perspective to the phonological model of dyslexia. In essence, in line with the new understanding of the possible role of neuronal oscillations in speech perception (Luo and Poeppel, 2007; Poeppel et al., 2008), Goswami (2011) suggests that deficits in syllabic perception at delta/theta (4–10 Hz) frequencies form the critical basis for the reading impairment in DD. However, the TSF could be applied to the various stages of processing within the visual system as well, prior to the information entering the phonological processing stage. This leads one to consider how oscillatory activity may influence visual processing. The following account proposes a mechanism that underpins reading, based upon recent neurophysiological demonstrations of how visual cortical areas communicate with each other and the biophysical limitations surrounding such communication. It leads with an introduction to how these factors influence a function long established in phylogeny, namely visual attention, which could hold the key for our ability to read.

## The role of top-down feedback signals from parietal cortex in serial visual search

**Figure 1:**
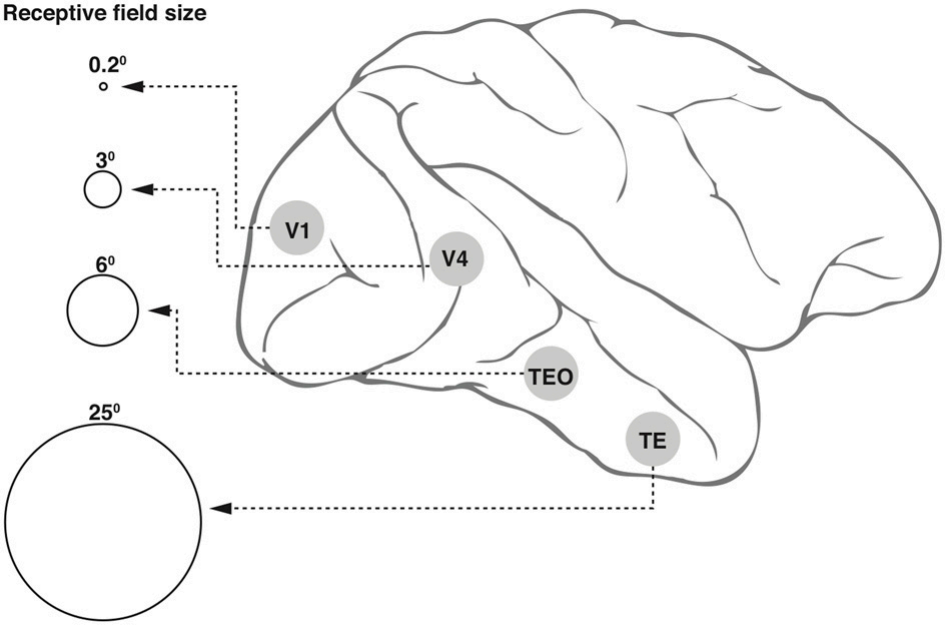
**Receptive field (RF) sizes along the ventral cortical stream in the primate**. While the degree of complexity of processing may increase, the RF size at any one eccentricity also increases dramatically along the various cortical areas from V1 into the temporal pole. The circles shown in the figure are not drawn to scale, but the numbers above the circles indicate approximate relative sizes of the RF diameters.

An alternative to the phonological basis for dyslexia has been the suggestion that the core deficit is in visuo-spatial attentional mechanisms that may be crucial to reading (Ans et al., 1998; Vidyasagar, 1999, 2001; Facoetti et al., 2000; Valdois et al., 2004; Pammer and Vidyasagar, 2005; Vidyasagar and Pammer, 2010). A number of studies have shown significant impairments in visual attention in children with DD (Casco and Prunetti, 1996; Hari et al., 1999; Vidyasagar and Pammer, 1999; Facoetti et al., 2000, 2008; Facoetti and Molteni, 2001; Bednarek et al., 2004; Kinsey et al., 2004; Valdois et al., 2004; Strasburger, 2005; Bosse et al., 2007; Roach and Hogben, 2007; Solan et al., 2007; Dhar et al., 2008; Jones et al., 2008; Kevan and Pammer, 2008; Bosse and Valdois, 2009; Facoetti et al., 2010; Ruffino et al., 2010). However, why should such a deficit in spatial attention cause dyslexia? This leads one to consider what may be the neural processes that nature has evolved that humans exploit in reading and what may be the neurophysiological constraints that these processes impose on our reading abilities. It has been proposed (Vidyasagar, 1999) that reading uses a circuit that the visual system had evolved to deal with the common problem of recognizing a target in a cluttered world. The visual system faces two major challenges in real visual scenes that are usually populated by myriad objects of different sizes, forms, colors, etc. These are:

1. Neurons in the ventral cortical stream that mediate object recognition exhibit progressively larger receptive fields (Figure 1) but yet display position invariance, i.e., they respond to the optimum stimulus, say a face, a car, or a letter, irrespective of their location within the large receptive field (Boussaoud et al., 1991). This and the various other invariances such as for retinal size and angle of view constitute an essential property for any object recognition system, so as to avoid the combinatorial problem of having neurons specific for a feature or object for every possible location, viewpoint, etc. However, the loss of location information will be an impediment in a number of situations, including that of reading a text, since the spatial sequence of letters within a word is vital for word recognition.
2. Different attributes of an object, such as color, form, depth, movement, etc are processed in separate cortical areas and there are over 30 of these in the primate cerebral cortex (Felleman and van Essen, 1991). How can these attributes be bound together so that the objects can be correctly identified?

One widely accepted solution to this double conundrum, supported by a long and continuing series of experiments using *visual search* paradigms, is that a spotlight of attention highlights one location at a time and the attributes of each object get bound by the coincident processing of its features (Treisman and Gelade, 1980; Treisman, 1988). The neuronal basis of this solution is likely to be a feedback from the parietal cortical region back to earlier visual areas, area V5/MT and still further back to V1, from where the two cortical streams originate (Vidyasagar, 1999). Many studies have now identified attentional facilitation of discrete locations in area MT (Saalmann et al., 2007) and the primary visual cortex (Vidyasagar, 1998; Brefzynski and DeYoe, 1999; Gandhi et al., 2001; McAdams and Reid, 2005; Simola et al., 2009). When only a small location of the visual scene on the primary visual cortex, pertaining to say one object, gets preferentially highlighted for a short period, the signals from this area will lead to unambiguous object identification despite the large receptive fields of neurons in the ventral stream; also, the different attributes of the object get bound together due to the coincidence of activation in different areas that process each of these attributes.

## Visual search and reading

The earliest scripts in recorded history are only about 6000 years old and many human communities did not have any written language until recently but all have the capacity to read and write. For this reason, it has been repeatedly pointed out that, during reading, we are using or “recycling” neural circuits that evolved over hundreds of thousands of years for other reasons (e.g., Vidyasagar, 1999; Dehaene and Cohen, 2007). What could these circuits be? It is now well established that the Visual Word Form area (VWFA), that shows specific activation during reading and dubbed the “letterbox area” of the brain (Cohen and Dehaene, 2004; Dehaene, 2009; Dehaene and Cohen, 2011), is one of the subdivisions in the ventral occipitotemporal region involved in object recognition (Puce et al., 1996; Kanwisher et al., 1997; Ishai et al., 1999; Haxby et al., 2001; Malach et al., 2002). This happens to be an area that codes for visual images of a particular spatial scale and when humans learn to read, the VWFA seems to be responsible for stringing letters together into words. In fact, literacy training in childhood even causes this area to expand at the cost of adjoining areas of the ventral visual cortex that code for objects at other spatial scales (Dehaene et al., 2010). Given such impressive plasticity of this ventral region, the core deficit in DD is unlikely to be a lesion here, unless one finds comparable deficits in object recognition of all types, which is not the case. However, prior to the stage of word recognition at VWFA, there are many steps in visual processing which are all critical for reading. Beyond the stage of initial processing of elementary contours such as lines and edges, a vital hurdle to be overcome is the conundrum described in the previous section. Written text such as the one you are reading now is almost certainly the most frequent and crowded visual scene at a fine spatial scale that we are confronted with in modern civilization. How do we manage to identify each letter and combine them in the appropriate sequence to form words despite the limitations mentioned earlier? One solution that has been proposed (Vidyasagar, 1999, 2001; Vidyasagar and Pammer, 1999, 2010) was that during reading we exploit the same circuits and processes that we had evolved for visual search and object identification in a cluttered world. It was hypothesized that during the periods of eye fixation in reading a text, each lasting approximately 250 ms, feedback signals to V1 sweep a spotlight of covert attention across the letters of individual words and this temporal sequence of identified letters leads to their concatenation into the spatial sequence subsequent to their serial recognition in the ventral stream. Given this scenario, reading may well be the most challenging task for the visuo-spatial attentional mechanisms in a visual world dominated by the printed text and any deficit of these attentional resources can lead to the impairment in what is possibly one of the most sensitive of all our brain functions, namely reading.

Many studies had implicated a deficit in the magnocellular pathway in dyslexia (e.g., Lovegrove et al., 1980; Livingstone et al., 1991; Cornelissen et al., 1995; Eden et al., 1996; Stein and Walsh, 1997; Pammer and Wheatley, 2001; Solan et al., 2004). Such a deficit can potentially be the basis of the attentional impairment (Vidyasagar and Pammer, 1999), since the visual inputs to the dorsal stream, the putative driver of the attentional feedback, is dominated by magnocellular signals. However, the magnocellular deficit has not always been found to be associated with dyslexia as reviewed by Skoyles and Skottun (2004). This discrepancy in the literature is not entirely surprising, for two reasons: (1) A visuo-spatial attentional deficit and consequent reading impairment can be caused not only by a lesion in the visual magnocellular pathway, but also in the dorsal stream structures themselves or in the feedback pathways to the striate cortex. (2) For a magnocellular deficit to manifest as dyslexia, it needs to be at the critical visual field site, namely a small region near the center of the fovea where covert attention is used for letter identification as described above. Unless a magno deficit involves this region, a reading difficulty need not be expected. That almost none of the studies have paid attention to this confounder, could be a contributory factor to the discrepancy in the literature on the relationship between magnocellular dysfunction and the reading impairment.

## The role of neuronal oscillations in visual search and reading

If, as suggested above, top-down feedback is essential for serial letter recognition in reading, what could be the neuronal mechanism that makes such feedback modulation and parsing of letters possible? The answer to this question may come from the recent realization that oscillatory activity of neurons is fundamental for organizing and integrating information in the brain (for review, Buzsáki, 2006). Recent studies into the role of synchronized oscillations in visual attention (Saalmann et al., 2007; Gregoriou et al., 2009; Miller and Buschman, 2013), and speech perception (Giraud and Poeppel, 2012) provide an insight into how neuronal oscillations might also mediate the top-down feedback process in visual search and reading.

Humans analyze speech in essentially two integration windows: one at ~150 ms and above, i.e., at delta-theta band for syllabic segmentation and one at ~30 ms and below, i.e., at low gamma range for phonemic segmentation (Giraud et al., 2007; Abrams et al., 2009; Lehongre et al., 2011; Giraud and Poeppel, 2012). Processing of human speech indicates that weak gamma oscillation at rest (30–40 Hz) gets stronger with auditory input and individual neurons tuned for specific acoustic frequencies tend to fire more at a particular phase at each cycle (Giraud and Poeppel, 2012). In this scenario, acoustic energy concentrated in formants in the feedforward input at delta-theta range is itself conceived to lead to nesting of the higher gamma frequencies.

These sampling principles in the time domain with acoustic speech signals can be applied also to the visual system, which has to perform a spatiotemporal sampling of the visual input. While modulation frequencies in the delta-theta (3–10 Hz) band are crucial in speech perception for syllabic segmentation of the amplitude envelope, there is no reason at all for segmentation at these rates to be crucial for the visual system in deciphering individual letters or for that matter concatenating them into a word. There is no actual physical modulation at the syllabic level in the visual input arising from a text. The critical visual segmentation is primarily at the level of graphemes, which in English, are the alphabetic letters. Regular, periodic, segmentation at the word level is impractical since word lengths vary considerably. This is not the case with letters which usually occupy approximately the same physical space and can be sampled in a nearly periodic fashion by a spatiotemporal sampling process.

The one naturally occurring process in the brain that could be exploited for parsing the neuronal signals related to letters in a text that arrive at the primary visual cortex is in fact the top-down signals that are usually used for serially highlighting items in a visual search task. Such modulation of early areas is now known to be mediated by synchronized neuronal oscillations (Fries, 2005; Saalmann et al., 2007). In visual search, one can envisage a small group of cells in the posterior parietal cortex (PPC) oscillating in synchrony when they mediate focal spatial attention (Saalmann et al., 2007), either due to the relative saliency of the sensory input or in response to top-down signals from the prefrontal executive regions (Buschman and Miller, 2009). The frequency of the neuronal synchrony of the top-down signals from PPC have been identified to be 25–45 Hz (Saalmann et al., 2007) and the ones from prefrontal cortex to PPC to be 22–34 Hz (Buschman and Miller, 2007).

Oscillations can potentially either define the periods when and how often a set of post-synaptic neurons would fire in response to inputs that otherwise are too weak to reach threshold or enhance responses to a specific set of input signals, or do both. By the same token, the lack of oscillatory modulation of the membrane potential may by default act as suppression, since it would require a much stronger input to lift the membrane potential to threshold. The feedback pathways are known to be excitatory in nature (Anderson et al., 2011), but local inhibitory connections could cause suppression of unattended stimuli (Miller and Buschman, 2013).

**Figure 2:**
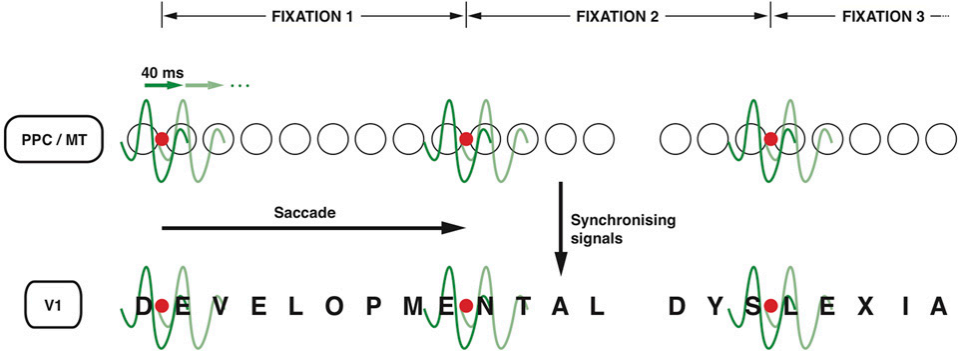
**Neuronal oscillations underlying the spotlight of attention that enables sequential letter processing during reading**. A wavelet of low gamma frequency (say around 25 Hz, i.e., with a period of 40 ms) sweeps across the retinotopic map in posterior parietal cortex and MT leading to sequential shift of the locus of top-down facilitation in V1 through interareal synchrony. The letters in the bottom row (DEVELOPMENTAL DYSLEXIA) represent the bottom-up sensory signals in V1 corresponding to each letter of the text that is being read. The fine grain representation of the visual world in V1 preserves the form of each letter, even though they are not coded as “letters” in V1. The circles in the middle row represent the loci of activities in LIP and MT that code for letter locations in the text. The letter representations highlighted by each subsequent cycle of the gamma wavelet are hypothesized to be processed and recognized individually and in sequence in the ventral stream. Only two cycles are shown for each fixation but they are presumed to sweep across the letters away and to the right of the fixation point to cover 7–8 letter-spaces during each fixation. The red dot represents the point of eye fixation. The top row indicates the periods of fixation, each lasting 250–300 ms.

It is not unlikely that the top-down signals from the PPC to MT and further to V1 (Vidyasagar, 1998; Brefzynski and DeYoe, 1999; Gandhi et al., 2001; McAdams and Reid, 2005) that are essential for serial visual search might also be exploited for reading. The same oscillatory nature of the modulation can be used for scanning the printed text during reading and process one letter (or two) at a time. One could conceive of each cycle of an oscillating wavelet in the low gamma range highlighting one of the salient locations on the visual field representation (Figure 2). This permits discrete chunks of information, just from one location or object, to be processed concurrently in the ventral stream for perceptual binding and object identification. For this to work in serial visual search, one would expect the salient locations on the parietal priority map to be activated in sequence with each cycle of a gamma wavelet switching to a different spatial location. There is evidence for this from the work of Buschman and Miller in macaques (2009; 2010), which showed spatial attention in a serial search task being directed by FEF in the frontal cortex with the fronto-parietal synchrony serially activating object locations in LIP. In fact, monkeys were found to shift their covert attention every 40 ms and the neural correlate of such shifts were observed in the oscillations of local field potentials in the FEF synchronized to 25 Hz. In learning to read, I suggest that the fronto-parietal network gets trained to do a similar serial, but *spatially sequential*, switching of top-down focal attention signals.

A consequence of the above framework is that any sampling process that humans use for reading an alphabetic text such as English is likely to have a temporal frequency roughly related to the rate at which readers sample individual letters or small groups of letters (say, in twos or threes as most words have upwards of two or three letters in them). I propose that if reading exploits the same mechanisms that we employ for visual search, both reading speed, and print sizes would fall within limits that are ultimately determined by the low gamma frequency range used by the visual search mechanism for top-down gating of visual signals arriving at V1. Thus, the speed of both these processes—visual search and reading—measured as items or characters (alphabets) processed in unit time—will be within the major frequency band of the synchrony between these areas during periods of focal spatial attention. Most common visual search paradigms yield a slope of 20–45 ms/item depending upon task demands (e.g., Wolfe et al., 1998; Wolfe and Horowitz, 2004), whose reciprocal, namely frequency, in cycles per second is between 22.2 and 50 Hz. This is in fact very similar to the range (25–45 Hz) of low gamma frequencies that the parietal cortex has been shown to use for top-down modulation of early visual areas (Saalmann et al., 2007).

With most instances of visual search, the main immediate task may be only object identification, but in the case of reading, the cognitive load is considerably beyond simple identification of letters and words. After the letters are concatenated into words, the words need to be semantically interpreted, strung together in to a sentence and the overall meaning of sentences and the passage comprehended. Furthermore, there is the necessity of having to proceed along a specific spatial gradient (left to right in the horizontal direction in English) to the exclusion of all other directions. Therefore, for a function such as reading that is forced to use an evolutionarily older process (visual search) whose sampling bandwidth is limited to a range of 22.2–50 Hz, it would be best to use the slowest possible speed within that range, thus, parsing letters at the lower end of this range to prevent the subsequent cognitive processing stages from being overloaded.

How well are these predictions borne out by data on reading speeds? In a major study (Rubin and Turano, 1992), the average reading speed was found to be in fact a maximum of 303 words/min. This translates, in English which has an average of 4.5 letters to a word, to about 23 alphabetic characters per second (23 Hz) or nearly 44 ms per letter. This is almost precisely the lower end of the neuronal oscillation range for the top-down signals from PPC that mediate focal spatial attention (Saalmann et al., 2007). A further testable prediction from the above framework is that the variation in reading speed seen in the population may roughly reflect the variation between individuals in the speed of visual search. In fact, recent experiments (Verghese et al., under review) indicate a significant positive correlation between these two variables. Valdois and colleagues, investigating further their earlier finding that reading performance was influenced by visual attention span (Bosse and Valdois, 2009), also found that reading speed was affected by only one component of visual attention capacity, namely visual processing speed (Lobier et al., 2013). These observations follow directly from the above theory that the speed determined by the gamma frequency oscillation is the essential rate-limiting step in reading.

## Physiological constraints on oscillations, visual search and reading

If visual search and reading are dependent on oscillating signals that mediate the essential cortical interactions, they are inevitably locked into a narrow range of speeds that are related to the low gamma range of the neural synchrony that underpins the interactions. In turn, there are compelling biophysical and physiological reasons why the frequency of oscillations themselves fall within a particular range (Buzsáki, 2006). Frequency of rhythmically discharging GABAergic neurons and the resonance properties of pyramidal cells determined by their time constants allow only a relatively narrow window of frequencies of oscillations that could facilitate within-area and between-areas synchrony (Hutcheon and Yarom, 2000; Markram et al., 2004; Economo and White, 2012). This may be the reason why the commonly encountered neuronal oscillations are in the gamma frequency range, 20–70 Hz, which resonate best with neurons, which all tend to have integration times between 15 and 50 ms. While within-area synchrony may be largely driven by relatively higher gamma frequencies, typically in the 35–85 Hz range, as for example for stimulus driven synchrony of V1 cells (Eckhorn et al., 1988), between-area synchronization is usually slower, being 25–45 Hz for the LIP (an area within the macaque PPC) to MT feedback (Saalmann et al., 2007). This may be related to the type of post-synaptic cells that receive the feedback, the local circuitry that could influence the active properties of the cells and their time constants. While the frequency of the feedback to V1 from area MT mediating focal attention has not yet been directly measured, we already know that the MT cells fire at 25–45 Hz during focal spatial attention (Saalmann et al., 2007).

**Figure 3:**
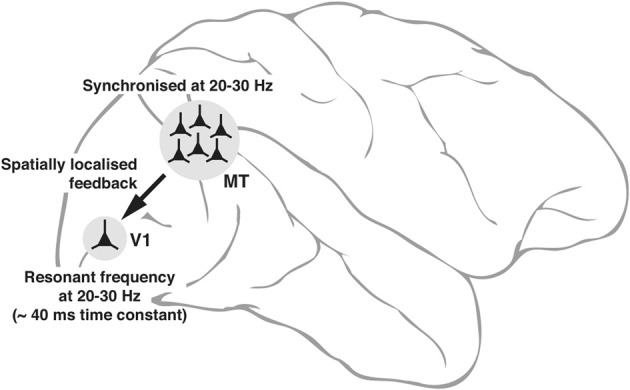
**Schematic diagram, explaining the neuronal basis of feedback oscillations**. Local network properties and biophysical parameters such as time constants of neurons determine the synchronizing frequencies of neurons in MT that provide feedback to V1 and the resonant properties of neurons in V1. It is suggested that a group of neurons representing a single location (of say, an object) in the parietal saliency map sending its signals via the corresponding group of neurons in MT can set the membrane potential of the spatially equivalent group of neurons in V1 (only 1 shown) to oscillate at its resonant frequency. Such an oscillation would facilitate the V1 neurons to respond to a sensory input more readily as the input signals ride on the top of the depolarizing crests in membrane potential.

The intrinsic properties of neurons—some of them subject to dynamic changes, such as conductance of ion channels and others not subject to changes such as the size of the cell itself that sets the outer limits to input resistance—can potentially play a fundamental role in the emergence of neuronal oscillations and to what frequencies in the input signals that the neuron will resonate most. These variables are reflected in the range of low gamma oscillations seen in interareal synchrony. Figure 3 is a putative model of the top-down modulation of V1 activity by a feedback from area MT at low gamma frequencies, whose range is determined by the resonance properties of the post-synaptic V1 cells and the circuitry they are embedded in.

While there are a number of cortical areas involved in reading such as the VWFA in the ventral cortex (for reviews, Dehaene, 2009; Wandell, 2012), there have been few electrophysiological studies so far that have identified the frequencies that mediate cortical interactions between these areas. The account presented in this paper has largely concentrated on the feedback within the dorsal stream for two reasons. First, there is relevant electrophysiological data available that provide us with some real numbers. Second, since the neural process mediating visual search is essential for parsing of letters in a text and determines reading speed, the neuronal oscillations in the dorsal feedback are likely to have a more decisive influence on the speed of orthographic processes than the oscillatory activities within the ventral stream.

## Synchronized oscillations and developmental dyslexia

There has been much controversy recently whether the core deficit in dyslexia is in phonological processing or in visuo-spatial attention (for reviews, see Ramus, 2003; Vidyasagar and Pammer, 2010; Goswami, 2011). Though there are profound phonological deficits in most cases of DD, the causality has not been established (Castles and Coltheart, 2004; Vidyasagar and Pammer, 2010; Vidyasagar, 2012), whereas there are now many studies claiming visual attentional deficits as the critical causative factor (e.g., Vidyasagar and Pammer, 1999; Facoetti et al., 2000, 2008; Facoetti and Molteni, 2001; Valdois et al., 2004; Bosse et al., 2007; Kevan and Pammer, 2008; Bosse and Valdois, 2009). In an attempt to anchor the aetiology of dyslexia in basic neurophysiology, Goswami (2011) attributed the phonological deficits and by inference the reading impairment to possible alteration of the syllabic sampling of speech (Goswami, 2011). The nesting of phonemic sampling at the low gamma rates of around 20–30 Hz within the low delta/theta syllabic frequency of around 4 Hz implies that altered sampling at syllabic rates could be the basis of the poor sensitivity of dyslexic children to low frequencies in the amplitude modulated acoustic input (Lorenzi et al., 2000). However, in a recent study of auditory steady state cortical responses (ASSR) to an amplitude modulated noise spectrum measured using magnetoencephalography and MRI (Lehongre et al., 2011), a significant deficit was seen in dyslexic subjects only for low gamma (phonemic) frequencies (20–30 Hz) in the left planum temporale. This appears at odds with the idea of a core deficit in syllabic (delta/theta) frequencies suggested by Goswami (2011), unless the low frequency transitions reset the neuronal activity occurring at gamma frequencies as suggested for selective attention by Schroeder and Lakatos (2009).

The sensory stimulus for the study by Lehongre et al. (2011) was acoustic, but I suggest that the deficit may be one that is more general across other modalities as well and the use of visual stimuli might expose deficits in visual areas, particularly those involved in visuo-spatial attention. As mentioned earlier, the critical sampling rate for visual search is indeed in the same range of low gamma frequencies and not the much slower delta/theta rate, which in fact shows no deficit in the study by Lehongre et al. (2011). The sampling rate that is used by the visuo-spatial attentional process for sequential reading of letters in a word is also the low gamma rate. The core deficit in DD may thus be an impairment in sampling at this rate. The deficits in speech perception and phonological processing may be a consequence of poorer sampling of the acoustic signals, but the reading deficit itself may be a consequence of similar impaired sampling at low gamma frequencies in the visual domain. In this scheme, the essential deficit in dyslexia is entirely within the visual system. The phonological deficits occur in parallel and to some extent they could also be partially the result of poor orthographic processing.

It should be noted that the sampling rate that we generally use for reading is not one that evolution had selected for reading but one that had possibly been selected for visual search and as noted above, this range is itself under certain constraints. However, reading comprehension, unlike reading speed, depends upon the working memory and cognitive capacities of the individual and the demands placed on them by the semantic content of the text. It is thus, most likely that as one learns to read, the brain uses the speed at the low end of the gamma range used for visual search (namely around 20–25 Hz), so that sufficient time can be spent on each grapheme and the subsequent processing stages. It is possible that due to the biophysical and network parameters that determine the limited range of frequencies for neuronal oscillations, the rate that one uses for reading may differ between individuals and this may explain the variance seen in reading speeds. Every reader ultimately gets “locked” into a particular narrow range of speeds, which makes slower or faster parsing of letters in a text inefficient. For complex material, the sampling rate one is locked into may turn out to be “too fast” for some. In fact, adult dyslexics, who are “compensated” may actually have a better reading comprehension scores even though their reading speeds will be slower than controls. On the flip side, fast readers (and hyperlexics), who are essentially “locked” into this higher speed, may have a gamma range for top-down modulation that is shifted toward higher frequencies, as indeed seen in their faster visual search (Verghese et al., under review). This shifted range will enable faster reading, but reading comprehension will be compromised due to overloading of the subsequent working memory and semantic stages.

The basic deficit in DD may be either an impairment (e.g., lower amplitude of oscillation) of the sampling at low gamma frequencies that affects visual search and reading and/or a slowing down of this rate. A change in amplitude or frequency of the synchronized oscillations is likely to affect the efficiency of modulation of V1 by the top-down feedback. Lower power will lead to poorer facilitation of sensory signals arriving in V1 and alteration in frequency would lead to a mismatch with the resonant frequencies of the V1 cells with subsequently poorer oscillations of the membrane potential. In either case, reading speed will be affected, but when there is some slowing down of the sampling rate, reading comprehension may in fact be better.

One interesting observation made by Lehongre et al. (2011) was that the dyslexics do not always show a general slowing of temporal processing as originally proposed by Tallal (1980). In fact the dyslexic subjects also show an increase in the 40 Hz activity in the right hemisphere to the acoustic stimuli. However, in the scheme proposed here, both slower and faster sampling would have consequences—slower by reducing reading speed and faster by overloading the working memory capacity and the time needed for semantic interpretation of the text.

Buzsáki (2006) has argued that the oscillating frequency of a cluster of neurons will be slower when the spatial extent of the group is larger. This has implications for the framework proposed here that involves a discrete moving spotlight of attention that is represented by a spatial sequence of small groups of neurons on the parietal priority map, that each fire in synchrony. Thus, if the spotlight of attention is larger and spans more than one letter at a time (as it could happen with experienced readers who may identify sets of two or even three letters at each gamma cycle), the rhythm may be slower. Thus, the reading speed may not be very much faster with a larger span, leading to a fairly narrow range of optimum font sizes in print. In fact, the distribution of print sizes in many types of publications all fall within a range that was indeed found optimal for reading (Legge and Bigelow, 2011).

It should not, however, be interpreted that the above scheme discounts the role of delta and theta frequencies that have been suggested as being critically important in reading and for speech recognition (Goswami, 2011; Power et al., 2012). In fact, the saccadic eye movements in reading occur on average at about 4 Hz, i.e., a saccade approximately every 250 ms (Rayner, 1998). One could argue that the sensory inputs get parsed at this rate, which is close to the word and syllabic reading speeds, a frequency range that has been implicated in the aetiology of DD (Goswami, 2011). However, the critical information processing essential for decoding a printed text is the parsing and identification of letters, which for most readers occurs at low gamma rates. In fact, this process may itself determine the saccade frequency. The decreasing strength of covert attention at increasing distance from the center of the fovea and the individual's preferred gamma frequency will limit in time and space the number of letters that can be sequentially identified during a single period of fixation and can even trigger the subsequent saccade. That the efferent oculomotor signals lead to saccades at the delta/theta frequency during reading does not imply that the afferent stream also gets segmented at the same rate for purposes of detailed sensory processing. Thus, the critical parsing of the afferent signals for grapheme recognition may be restricted largely to the low gamma range.

Low frequency oscillations in human cortical areas have also been reported in a number of other situations, for example with regard to sustained visual attention (Busch and VanRullen, 2010), speech recognition (Giraud and Poeppel, 2012; Power et al., 2012), auditory syntactic processing (Schmidt-Kassow and Kotz, 2008), and modulation of visual awareness (Mathewson et al., 2012). Taken together, these studies suggest the existence of a number of different time scales at which neural oscillations are linked to cognitive processes, ranging from the delta frequencies in decoding speech (Giraud and Poeppel, 2012) to the 12 Hz seen with visual awareness (Mathewson et al., 2012). As remarked earlier, the range of oscillating frequencies depend upon the network dynamics of the respective circuitry and the resonance properties of the cells in the network. Unlike the above instances, in the case of reading, the critical frequency used in specific decoding of the text may be the low gamma frequencies. These frequencies are known to be used in shifting the loci of attention in visual search tasks (Buschman and Miller, 2009, 2010) and are also impaired in DD (Lehongre et al., 2011).

It is worth stressing that reading a text is a very different process from speech recognition. Speech decoding is dictated by external inputs and has to be dynamic and flexible, occurring not only at multiple time scales (syllabic and phonetic), but also at the different frequencies that the speaker outputs, with the listeners having little choice in the sampling required of them. In reading this is not a problem, since the rate that is most optimal for the reader's perceptual and cognitive systems can be employed by every individual reader without any external constraint. Thus, the temporal characteristics associated with speech recognition need not necessarily determine the processing speeds of the visual system in the case of reading.

## Predictions of the scheme

A few testable predictions that specifically follow from the scheme outlined in this paper are summarized below:

1. Reading speed will show a positive correlation with the speed of visual search.
2. Reading speed will also show a positive correlation with the frequency of interareal synchrony between the dorsal stream and primary visual cortex.
3. Those with slower reading speeds, including many compensated adult dyslexics, will have better reading comprehension than faster readers.
4. As each individual is locked into a narrow range of sampling, one cannot force oneself to a slower reading speed by parsing letters more slowly, without seriously affecting reading comprehension.
5. In DD, the use of periodic visual stimuli will reveal an impairment in the low gamma frequencies in the visual cortical areas, similar to that seen in auditory areas with acoustic stimuli.

At this juncture, one might attempt to predict the consequences of the scheme for non-alphabetic languages such as Chinese. Even though in Chinese, there are only 1.5 characters per word on average (Sun et al., 1985), as against 4.5 letters to a word in English, it does not follow that the reading speed would be three times faster and it is not. The approximately 6000 Chinese characters cannot be taken as equivalent to the set of 26 alphabetic characters in English. A more appropriate level of parsing is by radicals which constitute a smaller set of 214 elemental units that go to form any one of the 6000 characters. As there are on average 2.6 radicals to a character (Shi et al., 2003) and the reading rate in horizontally written Chinese is 580 characters per minute (Sun et al., 1985), the speed of parsing would be ca. 40 ms/radical (25 Hz). This is in the same ballpark as in English (44 ms/letter on average).

The above argument can be extended also to difficult visual searches, where search speeds can be much slower, i.e., much longer than the 40–44 ms/item indicated above for reading. When searching for targets that are more complex than conjunction of elementary features such as lines and colors as in the case of a face in a crowd, a car in parking lot, or a book on a cluttered desk, the situation is more akin to that of reading Chinese characters. Thus, a process of parsing at low gamma frequencies across the details of each complex object would yield larger slopes per object. Unless there is a unique feature in the target that can lead to pop-out, complex objects would yield larger search slopes proportionate to the number of elementary features that they are composed of. However, parsing of these elementary features would *always* occur at the same low gamma frequency. This is a strong and testable prediction from the scheme proposed here.

## Conclusion

The development of writing and reading was a cultural programme in human history that happened to exploit a mechanism that had evolved for covert serial visual search. Top-down signals from a fronto-parietal network that uses neuronal synchrony for interareal attentional gating have been found to function at low gamma frequencies, with each cycle of the oscillation shifting to a different location during serial search. It is proposed here that this same mechanism is used for sequential scanning of individual letters during reading. This explains the fact that the reading speed of graphemes in a text is also in the same gamma range. This is consistent with the findings that with acoustic stimuli, there is an impairment of oscillations in the low gamma range in DD (Giraud and Poeppel, 2012).

Most genetic markers of DD seem to be involved in development of cortical laminae and migration of neurons (for review, Paracchini et al., 2007) and consistent with these, morphological changes in sizes of neurons and cytoarchitecture have been found in many regions of the brain including the visual system (for review, Galaburda and Livingstone, 1993; Galaburda, 1994). One important consequence of such changes in cell morphology and circuitry will be a change in the resonance properties of neurons and thus, the frequency and amplitudes of neuronal oscillations in the brain. Mild impairments of such brain rhythms may not affect most behavioral functions except those that are most sensitive to the disruption. As argued earlier, reading is one of the most challenging tasks in present civilization for visual attentional mechanisms, as the parsing of the text is done within a narrow spatiotemporal range. Thus, with any mild impairment of the attentional mechanisms, reading may be compromised, while for most other cognitive functions which also use top-down attentional processes, the deficit may not be so disabling.

### Conflict of interest statement

The author declares that the research was conducted in the absence of any commercial or financial relationships that could be construed as a potential conflict of interest.
